# Genetic parameter estimates and selection gain for multiple traits in white Guinea yam (*Dioscorea rotundata*) in Ghana

**DOI:** 10.1007/s10681-025-03467-x

**Published:** 2025-02-06

**Authors:** Kwabena Darkwa, Emmanuel Amponsah Adjei, Emmanuel B. Chamba, Alhassan Sayibu, Isaac Kodzo Amegbor, Freda Ansaah Agyapong, Ziblila Sayibu, Ibrahim Sayibu, Martain Kangmennaang, Muazu Issifu, Paterne A. Agre, Patrick Adebola, Asrat Asfaw

**Affiliations:** 1 CSIR – Savanna Agricultural Research Institute, Tamale, Ghana; 2 Department of Plant Breeding, Faculty of Agriculture and Natural Sciences, University of the Free State, P.O. Box 339, Bloemfontein, South Africa; 3 Department of Agricultural Biotechnology, Faculty of Agriculture Food and Consumer Sciences, University for Development Studies, Tamale, Ghana; 4 International Institute for Tropical Agriculture (IITA), Ibadan, Nigeria; 5 Present Address: J. Agribusiness Services Limited, Tamale, Ghana

**Keywords:** Genetic gain, Heritability, Selection index, White Guinea yam, Genetic correlation, Half-sib breeding

## Abstract

Quantifying selection gains enables a more targeted assessment of breeding program effectiveness, highlighting opportunities for strategic improvement and optimized genetic advancement in white Guinea Yam. This study assessed genetic parameters and gain for key traits in a white Guinea yam (*D. rotundata*) breeding population. A total of 81 genotypes were evaluated for two seasons using a 9 × 9 lattice design with three replicates. Data was collected on yam mosaic virus disease severity, tuber yield and tuber dry content for genetic analysis. Broad sense heritability was generally high (> 60) for most of the traits. At the same time, the corresponding genetic advance as a percentage of the mean was exceptionally high (30.28–93.96%) for tuber yield, tuber flesh oxidation, average tuber weight and number of tubers per plant, suggesting additive genetic effects. A multi-trait selection index of the 5% highest performing genotypes revealed positive genetic gain for plant vigour, tuber length, and width, tuber weight per plant, average tuber weight and fresh tuber yield. The genetic gain was, however, negative for tuber dry matter content compared to the check varieties, necessitating a modification of the current breeding scheme such that post-harvest food quality is not sacrificed for tuber yield. Ranking of the breeding lines based on the multi-trait selection index identified four lines (TDr1700004_014, TDr1700004_113, TDr1700001_112 and TDr1700002_090) with high genetic merits for all the economic traits. These lines can be used as potential trait progenitors and evaluated further for possible release as new varieties. Our results decipher the genetic control and provide an overview of the performance of the breeding program for key traits in white Guinea yam.

**Supplementary Information:**

The online version contains supplementary material available at 10.1007/s10681-025-03467-x.

## Introduction

Yam (*Dioscorea* species) is an important food security crop cultivated extensively in West Africa. Over 600 species comprise the genus *Dioscorea*, of which eleven are mostly grown for food in the tropics and sub-tropics (Degras 1993). The White Guinea yam (*Dioscorea rotundata)* originated from West Africa and is the most important species for cultivation and consumption (Lebot 2009). The crop has socio-cultural relevance and is integral in many key life ceremonies (Obidiegwu and Akpabio 2017). Yam is consumed in serval forms, including pounded, boiled, porridge, flour and chips. Yam is a good source of carbohydrates, proteins, minerals and vitamins, as well as several bioactive compounds such as phenolics, flavonoids, allantoin, dioscin, dioscorin, diosgenin, polyphenols, tannins, hydrogen cyanide, oxalate, saponin and alkaloids with pharmaceutical potentials (Obidiegwu et al. 2020).

The “yam belt” of Africa, spanning Cote d’Ivoire, Ghana, Togo, Benin Republic, Nigeria, and Cameroon (Coursey 1976) accounts for 95% of the global yam production of 88.3 million tons on 10.4 million hectares (FAOSTAT 2022). Nigeria alone accounts for 69.3% of the global output, followed by Ghana with 10.7 million tons, representing 12.1%. Among the three leading yam producers in the world, Ghana recorded above average yield per hectare of 18.4 tons, with Nigeria and Côte d’Ivoire producing 8.2 and 5.4 tons, respectively, which is below the global average of 8.5 tons per hectare. There is a general decline in the productivity of yam in the major producing countries. The annual increase in production is primarily driven by an increase in the area under production rather than productivity per unit area (Danquah et al. 2022).

Yam productivity remains between 12.5 and 33% of the estimated potential yield due to several biotic and abiotic constraints, alongside post-harvest challenges (Frossard et al. 2017; MoFA 2019; Danquah et al. 2022). Key constraints include diseases and pests such as yam anthracnose (*Colletotrichum gloeosporioides*), yam mosaic virus, nematodes (*Scutellonema bradys* and *Meloidogyne* spp.), and tuber rot. Other factors include low-yielding landraces, declining soil fertility, drought, and heat stress induced by climate change (Obidiegwu and Akpabio 2017). The prevailing trend of area expansion to increase yam production is not environmentally friendly and inherently unsustainable. Therefore, crop improvement strategies are urgently required to increase yam productivity per unit area to meet the ever-increasing demand for both local and export markets.

Genetic improvement is a key sustainable way to systematically address these production constraints. Yam breeding is inherently challenging due to the crop’s biological and genetic complexities. These include dioecious reproductive biology, shy flowering, high heterozygosity, varying ploidy levels, and a low multiplication ratio in vegetative propagation (Asfaw et al. 2019; Mondo et al. 2020). These challenges necessitate innovative approaches to improve yam breeding programs. Yam breeding requires the deployment of a comprehensive approach integrating market insights and precise product profiles to align breeding objectives with diverse market demands. This involves multifaceted parent trait profiling, strategic mate pairing to leverage complementary genetics, and rigorous testing through on-station and on-farm trials to select superior individuals, ultimately delivering impactful varieties to end-users (Asfaw et al. 2024).

Achieving genetic gain remains central to the success of yam breeding programs. Selection strategies based on a narrow set of traits are less effective due to potential trade-offs and unintended consequences on secondary traits (Olivoto and Nardino 2021). Therefore, breeding efforts must adopt a multi-trait selection approach to deliver varieties that meet the diverse needs of end-users. Studies have shown that genetic parameters, including heritability and genetic correlations, are critical in predicting selection gains and optimizing breeding schemes (Lamsal et al. 2017; Asfaw et al. 2017; Norman et al. 2021). Earlier studies in *D. rotundata* have demonstrated the feasibility of achieving selection gains across multiple traits, including tuber yield, dry matter content, and disease resistance (Asfaw et al. 2021; Norman et al. 2022). Despite these achievements, there is limited information on the variation of genetic parameters and selection gains across diverse genetic backgrounds of white Guinea yam emanating from both controlled and open-pollinated poly cross sources. Understanding this variation is essential for optimizing breeding strategies to achieve sustained genetic improvement. This study was therefore conducted to estimate genetic parameters and selection gains for key economic traits in white Guinea yam from diverse genetic backgrounds. This research provides insights into the heritable variation, trait relationships, and the potential for multi-trait selection and half-sib breeding in enhancing yam improvement efforts.

## Materials and methods

### Study area

**Fig. 1:**
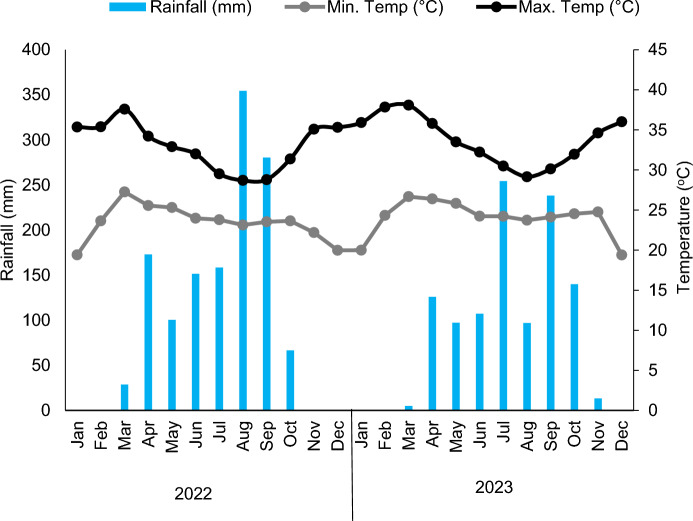
Average monthly minimum and maximum temperatures and average monthly cumulative rainfall at Nyankpala in 2022 and 2023

The study was conducted at the Council for Scientific and Industrial Research (CSIR)—Savanna Agricultural Research Institute (SARI) experimental station for two seasons (2022 and 2023). Nyankpala lies on Latitude 9°25′ 41″ N, Longitude 0°58′ 42″ W, Altitude 183 m asl. Northern Ghana lies within the Guinea Savanna Agroecology, with unimodal rainfall from May to October. Average monthly minimum and maximum temperatures were highest in March and lowest in December in both years. The average annual rainfall was 1313.2 mm in 2022 with no rainfall recorded in January, February, November and December and the highest was in August (354.3 mm) (Fig. 1). Similarly, there was no rainfall in January, February and December 2023. The highest rainfall of 254.3 mm occurred in July, with a lower annual rainfall of 1078.1 mm compared to 2022. The soils at the experimental site are Nyankpala series classified under Savanna Ochrosols, Plinthic Luvisols (FAO-UNESCO 1988; Adjei-Gyapong and Asiamah 2002). Soil samples from the experimental sites were collected and analyzed to assess the soil’s fertility status that supports the crop’s growth and development as described by ISRIC/FAO (2002). The pH of the soils at the experimental sites was slightly acidic, organic carbon and nitrogen were higher in 2022, while phosphorus and potassium were higher at the 2023 site (Table 1). Temperature and rainfall conditions at the experimental sites are presented in Fig. 1.

**Table 1:** Soil analysis at the experimental sites

Soil parameters	2022	2023
pH (H_2_O 1:2.5)	5.80	5.54
Organic carbon (%)	1.443	0.975
Nitrogen (%)	0.136	0.086
Phosphorus (mg/kg)	15.435	16.8
Potassium (mg/kg)	72.0	78.0

### Study materials and trial design

The preliminary performance stage trial consisted of 71 white Guinea yam (*Dioscorea rotundata*) genotypes and 10 checks. Sixty-five (65) of these test lines emanated from controlled crosses involving 4 elite female and 5 elite male parents at the International Institute of Tropical Agriculture (IITA), Ibadan, Nigeria and the remaining 26 were from open-pollinated crosses from 7 local female landraces at the CSIR—Savanna Agricultural Research Institute, Nyankpala, Ghana (Supplementary Table 1). The ten checks included four improved varieties and six popular local (farmer) varieties. The 81 entries were arranged in a 9 × 9 triple lattice design in both seasons of evaluation. Each experimental unit comprised a single-row plot of three plants using inter and intra-row spacing of 1.2 m. Planting was done in May in both years on manually raised yam mounds. Yam setts weighing between 150 and 200 g were used as planting materials. Healthy whole yam tubers were cut into setts, and the middle portion was used for the experiment to maintain the uniformity of the planting materials. The setts were treated with a mixture of 70 g Mancozeb, 75 mL Chlorpyrifos and 10 L of tap water for 10 min and air dried under shade for 24 h before planting.

No fertilizer was applied. The experimental field was maintained weed-free throughout the growing season by ensuring regular hand weeding for optimal conditions for plant growth and development. At 8 months post-planting, the trial was harvested upon reaching full senescence, characterized by complete drying of leaves and vines.

### Plant traits measurement

Data was collected on growth, yield and disease parameters following the standard procedures outlined in the yam trait ontology (https://yambase.org/search/traits; Asfaw 2016).

Yam sett weight was measured using a sensitive electronic scale. Plant vigour was visually assessed two months post-planting using a 1–3 scale, where 1 indicated weak (small plants with few leaves and thin vines), 2 indicated medium (intermediate or normal), and 3 indicated vigorous (robust plants with thick vines and abundant foliage). Yam mosaic virus (YMV) disease severity was evaluated using a 1–5 ordinal scale, assessing the relative area of symptomatic plant tissue, where 1 (no visible symptoms), 2 (mosaic on most leaves), 3 (mild symptoms on few leaves), 4 (severe mosaic and leaf distortion), and 5 (severe mosaic, bleaching, and stunting). YMV severity was assessed at 60, 75, 90, 105, 135, 165, and 180 days after planting. Disease resistance was quantified using the area under the disease progression curve (AUDPC) values, calculated from YMV severity scores over time, as described by Forbes et al. (2014).

1 $$AUDPC = \mathop \sum \limits_{i = 1}^{n - 1} \left( {\frac{yi + yi + 1}{2}} \right)\left( {ti_{ + 1} - ti} \right)$$

where y*i* = disease severity score at the *i*th observation, t*i* = time (days) at the *i*th observation and n = total number of observations.

Harvested plant count and sett weight were recorded and used as covariates to adjust fresh tuber yield. Fresh tuber yield components were assessed, including tubers per plant, tuber weight per plant (kg plant^−1^), and average tuber weight (kg tuber^−1^). Total fresh tuber weight per plot was measured using an electronic scale, and fresh tuber yield per hectare (t ha^−1^) was extrapolated by multiplying plot yield by 10 and dividing by effective plot area. Tuber dry matter content (%) was determined by oven-drying 200 g fresh tuber samples at 70 °C for up to 72 h and calculating dry weight as a percentage of fresh weight. Tuber flesh oxidation intensity was visually scored 30 min after cutting using a 1–3 scale, where 1 = no oxidization, 2 = slightly oxidizing, and 3 = highly oxidizing. Tuber length and width were measured for three tubers per plot using a measuring tape.

### Data analysis

#### Analysis of variance

Data analysis was conducted using the META-R software version 6.0 (Alvarado et al. 2020). The linear mixed models used in META-R are implemented in the LME R-package (Bates et al. 2015) that estimates variance components using REML according to the model:

2 $$Y_{ijkl} = \mu + Year_{i} + Rep_{j} \left( {Year_{i} } \right) + Block_{k} \left( {Year_{i} Rep_{j} } \right) + Gen_{l} + Year_{i} \times Gen_{l} + Cov + \varepsilon_{ijkl}$$

where ${Y}_{ijkl}$ is the trait of interest, $\mu$ is the overall mean effect, ${Rep}_{j}$ is the effect of the *j*th replicate, ${Year}_{i}$ is the effect of the *i*th year, ${Block}_{k}\left({Year}_{i}{Rep}_{j}\right)$ is the effect of *k*th incomplete block within the *j*th replicate in the *i*th year, ${Year}_{i} \times {Gen}_{l}$ is the year by genotype interaction. All effects in the model were declared as random except the overall mean and covariate. The random assumption of the genotype effects enabled the estimation of Best Linear Unbiased Predictions (BLUPs) and broad-sense heritability. The genotypes were however regarded as fixed effects when calculating the Best Linear Unbiased Estimators (BLUEs).

#### Broad sense Heritability and genetic correlation

The broad-sense heritability for the combined analysis of the two years was calculated as:

3 $$H^{2} = \frac{{\delta^{2} g}}{{\delta^{2} g + \frac{{\delta^{2} ge}}{nYear} + \delta^{2} \varepsilon /\left( {nYear X nRep} \right)}}$$

where ${\delta }^{2}g$ = genotypic variance, ${\delta }^{2}\varepsilon$ = error variance, ${\delta }^{2}ge$ = genotype by environment (year) variance component, and nYear = number of years (environments) and nRep = number of replications. Heritability was classified as low (0–30%), moderate (31–60%) and high (> 60%) as suggested by Comstock et al. (1949).

The genetic correlation among the yam traits were estimated using variance component derived from the fitted model in Eq. 2. A separate model was fitted for trait *i,* for trait *j*, and for the sum of both traits *i* and *j.* The genotypic covariance between traits *i* and *j* was calculated as:

4 $$\delta_{gij}^{2} = \frac{1}{2}(\delta_{gi + j}^{2} - \delta_{gi}^{2} - \delta_{gj}^{2} )$$

where ${\delta }_{gi+j}^{2}$= the genotypic covariance of the sum of traits *i* and *j*, ${\delta }_{gi}^{2}$ = genotypic covariance of the *i*th trait, ${\delta }_{gj}^{2}$= genotypic covariance of the *j*th trait.

The genetic correlation between the trait was calculated as

5 $$P_{gij} = \frac{{\delta_{gij}^{2} }}{{\delta_{gi}^{2} \delta_{gj}^{2} }}$$

where ${\delta }_{gi}^{2} \text{and} {\delta }_{gj}^{2}$ are the square roots of genotypic variances of the ith and jth traits, respectively.

#### Mean performance of genotypes and traits

A one degree of freedom contrasts was employed to compare the means of the 10 checks and the means of the 71 breeding lines and the means of the 56 TDr lines and the 25 SDr lines using the CONTRAST procedure in Statistical Analysis System software version 9.4 (SAS 2014). A comparison was also made between the four highest (HIGH) and the four lowest (LOW) yielding lines, simulating a 5% selection intensity to dissect the dynamics of the various traits in these lines to identify the traits that had the highest effect on the change in yield. A comparison was made between the selection differentials of the HIGH and LOW lines, but these were proportional to the performance of the selected groups in the following generation; heritability being the coefficient of proportionality.

#### Genetic parameters

The magnitude of genetic parameters, including phenotypic variance, genotypic variance, environmental variance, genotypic coefficient of variation (GCV), phenotypic coefficient of variation (PCV), heritability and genetic advance were computed for the traits to assess the extent to which a trait would be amenable to selection. Phenotypic and genotypic variance were estimated as indicated by Burton and Devane (1953) and used to compute the phenotypic and genotypic coefficients of variation, according to Singh and Chaudhary (1985). The phenotypic coefficient of variation (PCV) and genotypic coefficient of variation (GCV) values were classified as postulated by Sivasubramaniah and Meron (1973), in which PCV and GCV values above 20%, between 10 and 20%, and below 10% were categorized as high, moderate and low, respectively. The magnitude of genetic advance (GA) to be anticipated from selecting the top 5% of the lines and GA as a percentage of the mean (GAM) were estimated and categorized as low (0–10%), moderate (10–20%) and high (> 20%) as given by Johnson et al (1955).

#### Multi-trait selection and genetic gain

In the selection and ranking of high-performing individuals for genetic enhancement and gain for multiple traits concurrently, the following additive selection index for an estimated genotypic value was implemented (de Figueiredo et al. 2012).

6 $$I_{i} = \mathop \sum \limits_{k = 1}^{n} \beta_{it} \times {\text{ wt }} \times { }\frac{1}{{\sigma_{gt} }}$$

where ${I}_{i}$ is the selection index of an individual *i*, ${\beta }_{it}$ is the BLUP estimated genotypic value of an individual *i* for trait *t*, *wt* is the economic weight or degree of importance for trait *t*, and ${\sigma }_{gt}$ is the genotypic standard deviation for trait *t.* The economic weights assigned were 0.20 for number of tubers per plant, 0.30 for tuber weight per plant, 0.30 for fresh tuber (t ha^−1^), 0.30 for average tuber weight (kg tuber^−1^), 0.20 for tuber dry matter content, -0.15 for tuber flesh oxidation and − 0.15 for yam mosaic virus disease severity score (Area under disease progression curve). The top 5% breeding lines were selected based on the selection index that combines multiple traits.

The genetic gain as a percentage of the population mean was computed to determine the expected genetic gain for the traits applied for the selection of superior lines according to the procedure proposed by Cobb et al. (2019).

7 $$\Delta G_{m} = \frac{{K {\text{x}} \sqrt {h^{2} } {\text{x}} \sigma_{a} }}{{\overline{x}}}x 100$$

where $\Delta G_{m}$ is genetic gain as percentage of population mean, K is standardized selection differencial, which is 2.06 at 5% intensity, $\sqrt{{h}^{2}}$ is the repeatability or precision of the additive genotypic value (selection accuracy), ${\sigma }_{a}$ is the genetic standard deviation for the trait, $\overline{x }$ is the population mean. The ${\Delta G}_{m}$ values less than 10% were categorized as low, 10–20% as moderate and value above 20% as high (Johnson et al. 1955).

The expected genetic gain for the studied traits were further determined with standard varieties as benchmarks using the equation:

8 $$\Delta G_{c} = \frac{{\overline{X}_{it} - C_{it} }}{{C_{it} }}x 100$$

where ${\Delta G}_{c}$ is the observed genetic gain as compared with the standard varieties, ${\overline{X} }_{it}$ is the mean BLUP values of the selected individuals for trait *t*, and ${C}_{it}$ is the BLUP values of the corresponding standard (local or improved) variety. ${\Delta G}_{c}$ was applied to estimate the genetic gain for best 5% breeding lines selected based on the multiple trait selection index.

## Results

### Variability in agronomic traits

The analysis of variance of the combined years’ data revealed significant (*p* < 0.001) genotype and genotype by year interaction effects for all the traits studied except for tuber length and tuber dry matter content, where the genotype by year interaction was not significant (Table 2). The main effect of year was significant (*p* < 0.01) for yam mosaic virus disease severity score (AUDPC), average tuber weight, tuber length, tuber width and fresh tuber yield.

**Table 2:** Mean squares of traits of white Guinea yam evaluated for two seasons in Northern Ghana

Trait	Mean squares						
	Year	Rep (year)	Block (year*rep)	Genotype	Genotype * year	CV (%)	Mean
Plant vigour	0.04 ns	0.25 ns	0.16*	0.60**	0.24**	13.65	2.42
YMV (AUDPC)	3443.14*	373.17 ns	552.73 ns	1331.47**	669.93*	11.1	189.05
Number of tubers per plant	0.84 ns	0.14 ns	0.17 ns	1.08**	0.36*	25.02	1.93
Average tuber weight(kg)	1.61*	0.54 ns	0.29 ns	1.52**	0.50**	29.43	1.74
Tuber weight per plant (kg)	0.04 ns	0.51 ns	0.29 ns	1.13**	0.59**	21.31	2.48
Tuber length (cm)	402.08**	4.41 ns	21.44 ns	112.95**	27.18 ns	13.43	34.1
Tuber width (cm)	12.92*	3.66 ns	3.41 ns	12.10**	7.73**	12.96	13.79
Tuber dry matter content (%)	0.28 ns	106.38**	12.83 ns	40.36**	6.25 ns	10.16	32.22
Tuber flesh oxidation	0.46 ns	0.009 ns	0.12 ns	3.32**	0.32**	24.74	1.59
Fresh tuber yield (t/ha)	52.78*	6.29 ns	9.34 ns	28.63**	13.03**	14.29	19.23

YMV, yam mosaic virus disease severity score; AUDPC, area under disease progression curve; CV, coefficient of variation

^*^significant at 5%, **significant at 1% probability level

A one degree of freedom contrast between the lines and checks revealed that the average of the 71 lines was significantly (*p* < 0.01) higher than the average of the 10 checks for tuber weight per plant, average tuber weight, tuber length, tuber flesh oxidation, and fresh tuber yield (t ha^−1^) (Table 3). The higher average tuber flesh oxidation of the lines is not in the desired direction as high values means susceptibility to tuber flesh enzymatic browning (Table 3). The mean of the checks was however higher than that of the lines for tuber dry matter content. The difference between the lines and checks was positive for plant vigour and negative for yam mosaic virus disease severity score (AUDPC), albeit not significant. The average of the lines was higher by 2.47 t ha^−1^ for fresh tuber yield, by 3.40 cm for tuber length, by 0.45 kg for tuber weight per plant, and by 0.32 kg for average tuber weight than the checks (Table 3). Conversely, the checks were higher in tuber dry matter content by 3.20% and by 18.05 for yam mosaic virus disease severity score (AUDPC). It is worthy of note that lower values of AUDPC are desirable as higher values indicate more susceptibility to yam mosaic virus disease.

**Table 3:** The difference between the 71 test lines and 10 checks for various traits of white Guinea yam genotypes evaluated for two seasons in Nyankpala, Ghana

Genotype	Mean square	§ ESTIM	Mean		Diff	% Increase
			Breeding lines	Checks		
Plant vigour	0.07 ns	− 0.18	2.44	2.21	0.23	10.46
YMV (AUDPC)	126.94 ns	− 7.68	187.32	205.37	− 18.05	− 8.79
Number of tubers per plant	0.50 ns	− 0.48	1.92	2.01	− 0.09	− 4.70
Tuber weight per plant (kg)	0.13*	0.25	2.52	2.07	0.45	21.89
Average tuber weight (kg)	0.13*	0.24	1.81	1.48	0.32	21.86
Tuber length (cm)	682.04**	18.14	33.97	30.57	3.40	11.13
Tuber width (cm)	19.63 ns	3.24	13.75	13.58	0.17	1.27
Tuber flesh oxidation	0.15*	0.26	1.66	1.11	0.54	48.86
Tuber dry matter content (%)	191.45**	− 9.16	31.82	35.02	− 3.20	− 9.13
Fresh tuber yield (t/ha)	38.18*	− 4.20	19.23	16.76	2.47	14.72

DIFF, difference between means for breeding lines and checks; YMV, yam mosaic virus disease severity score (AUDPC: area under disease progression curve)

NS, *, **, ***non-significant, significant at *P* ≤ 0.05, significant at *P* ≤ 0.01, and significant at *P* ≤ 0.001, respectively, §ESTIM, estimate of the magnitude of the difference in the means

A comparison of the TDr and SDr lines using a one degree of freedom contrast showed that the mean tuber length of the 56 TDr lines was significantly higher (*p* < 0.01) than the mean of 25 SDr lines by 1.34 cm (Table 4). On the other hand, the mean of the SDr lines was significantly higher than that of the TDr lines for tuber dry matter content by 5.09%. The average of the TDr lines was higher by 0.13 kg for tuber weight per plant, by 0.19 kg for average tuber weight, by 0.41 for tuber flesh oxidation, and by 2.71 t ha^−1^ for fresh tuber yield than the SDr lines even though not significant. For tuber width and yam mosaic virus disease severity score, the average of the SDr lines was higher than that of TDr lines.

**Table 4:** The difference between the TDr and SDr lines for various traits of white Guinea yam genotypes evaluated for two seasons in Nyankpala, Ghana

Trait	Mean square	§ ESTIM	Means		Diff	% Increase
			TDr lines	SDr lines		
Plant Vigour	0.071 ns	0.072	2.42	2.38	0.03	1.46
YMV (AUDPC)	126.942 ns	3.072	188.23	192.51	− 4.28	− 2.23
Number of tubers per plant	0.508 ns	0.194	1.910	1.98	− 0.08	− 3.81
Tuber weight per plant (kg)	0.137 ns	− 0.101	2.50	2.37	0.13	5.47
Average tuber weight (kg)	0.133 ns	− 0.099	1.83	1.64	0.19	11.34
Tuber length (cm)	682.048**	− 7.259	33.96	32.62	1.34	4.11
Tuber width (cm)	19.639 ns	− 1.298	13.66	13.89	− 0.23	− 1.66
Tuber flesh oxidation	0.159 ns	− 0.105	1.72	1.3	0.41	31.77
Tuber dry matter content (%)	191.451**	3.666	31.69	33.39	− 1.7	− 5.09
Fresh tuber yield (t/ha)	38.189 ns	1.682	19.76	17.05	2.71	15.87

Diff, difference between means for TDr lines and SDr lines, YMV, yam mosaic virus disease severity score (AUDPC: area under disease progression curve)

NS non-significant, *significant at *P* ≤ 0.05, **significant at *P* ≤ 0.01, §ESTIM, estimate of the magnitude of the difference in the means

Under 5% selection intensity, the top four high-yielding lines (HIGH), namely TDr1700004_014, TDr1700004_113, TDr1700002_114, TDr1700001_112 and the bottom four low-yielding lines (LOW), namely SDr1403022_156, SDr1403002, SDr1403068_058 and SDr1403003_048 were identified. The difference between the HIGH and LOW lines was significant for all the traits assessed except for tuber width and dry matter content (Table 5).

**Table 5:** The difference between the 5% highest (HIGH) and lowest (LOW) yielding white Guinea yam lines for various traits across two seasons of evaluation in Nyankpala, Ghana

Trait	Mean square	§ ESTIM	Means		Diff	% Increase
			High	Low		
Plant vigour	1.809**	0.003	2.79	2.08	0.71	34.07
YMV	13905.272**	− 0.241	176.70	233.26	− 56.57	− 24.25
Number of tubers per plant	6.964**	0.005	2.11	1.95	0.16	7.99
Tuber weight per plant (kg)	0.003**	0.001	3.98	1.35	2.62	194.10
Average tuber weight (kg)	3.416*	− 0.004	2.60	0.92	1.68	182.75
Tuber length (cm)	1451.172**	0.078	46.80	25.53	21.27	83.32
Tuber width (cm)	3.965 ns	− 0.004	15.36	13.39	1.97	14.71
Tuber flesh oxidation	8.340**	0.005	1.16	1.49	− 0.33	− 22.39
Tuber dry matter content (%)	1.144 ns	0.002	31.26	30.33	0.93	3.06
Fresh tuber yield (t/ha)	7.268**	0.005	31.36	11.76	19.60	166.75

Diff, difference between means for high lines and low lines, Ymv, yam mosaic virus disease severity score (AUDPC: area under disease progression curve)

NS, *, **, *** non-significant, significant at *P* ≤ 0.05, significant at *P* ≤ 0.01, and significant at *P* ≤ 0.001, respectively, §ESTIM, estimate of the magnitude of the difference in the means

The HIGH lines gave advantages of 166.75% for fresh tuber yield per hectare, 194.10% for tuber weight per plant, 182.75% for average tuber weight and 83.32% for tuber length (Table 5). Plant vigour, tuber dry matter content, tuber width and number of tubers per pant improved by 34.07%, 3.06% and 14.71% respectively, when compared to LOW lines. The average yam mosaic virus disease (YMV) severity score and tuber flesh oxidation intensity score were higher for the LOW lines by 24.25% and 22.39%, respectively, compared to the High lines. This indicates that the LOW lines had higher YMV disease severity (susceptible), and higher tuber flesh enzymatic browning (oxidation) than the HIGH lines.

### Genetic variability and broad-sense heritability

The genotypic variance estimates were higher than the corresponding environmental and genotype by location variance estimates for all the traits assessed (Table 6) indicating a higher influence of the genotype on the phenotypic expression of the traits.

**Table 6:** Estimates of variance components of traits of white Guinea yam genotypes evaluated for two seasons in Nyankpala, 2022 and 2023

Traits	Genotypic variance	Phenotypic variance	Environmental variance	Gen x Loc variance	Residual variance
Plant vigour	0.073	0.123	0.001	0.059	0.123
YMV (AUDPC)	248.046	386.007	31.954	128.031	443.670
Number of tubers per plant	0.211	0.284	0.011	0.074	0.219
Average tuber weight (kg)	0.423	0.506	0.008	0.074	0.272
Tuber weight per plant (kg)	0.222	0.330	0.001	0.125	0.275
Tuber length (cm)	20.797	25.583	8.250	2.835	20.213
Tuber width (cm)	1.436	3.263	0.000	2.594	3.183
Tuber dry matter content (%)	9.034	10.642	0.001	0.001	9.644
Tuber flesh oxidation	0.680	0.737	0.005	0.067	0.142
Fresh tuber yield (t ha^−1^)	15.047	18.610	0.047	3.733	10.175

YMV, yam mosaic virus disease severity score (area under disease progression curve), Gen × Loc, genotype by location

The genotypic coefficient of variation (GCV) ranged from low (< 10) to high (> 20) (Table 7). Yam mosaic virus disease severity, tuber width and tuber dry matter content had low GCV values, while high GCV estimates were observed for number of tubers per plant, average tuber weight, tuber flesh oxidation and fresh tuber yield. Plant vigour, tuber weight per plant and tuber length recorded moderate GCV estimates. Estimates of phenotypic coefficient of variation (PCV) were moderate for plant vigour, yam mosaic virus disease severity score, tuber length and width and tuber dry matter content, while number of tubers per plant, average tuber weight, tuber weight per plant, tuber flesh oxidation and fresh tuber yield gave high PCV. The small difference between the GCV and PCV signifies a higher contribution of the genotypic variance to the phenotypic variance hence a higher gain from selection is expected.

**Table 7:** Broad sense heritability, genetic advance, phenotypic and genotypic coefficients of variation of traits of white Guinea yam genotypes evaluated for two seasons at Nyankpala, Northern Ghana

Traits	Heritability	GA	GAM	PCV	GCV
Plant Vigour	0.59	0.25	10.42	14.56	11.23
YMV	0.64	16.24	8.57	10.37	8.31
Number of tubers per plant	0.74	0.59	30.68	27.62	23.78
Average tuber weight (kg tuber^−1^)	0.84	1.01	57.21	40.20	36.79
Tuber weight per plant (kg plant^−1^)	0.67	0.52	21.28	23.32	19.12
Tuber length (cm)	0.81	6.69	19.94	15.08	13.59
Tuber width (cm)	0.44	0.69	5.05	13.15	8.72
Tuber dry matter content (%)	0.85	4.78	14.85	10.13	9.33
Tuber flesh oxidation	0.92	1.49	93.96	54.02	51.88
Fresh tuber yield (t ha^−1^)	0.81	5.73	30.28	22.80	20.50

GA, genetic advance, GAM, genetic advance as percentage of population mean, PCV, phenotypic coefficient of variation, GCV, genotypic coefficient of variation, YMV, yam mosaic virus disease severity score (AUDPC: area under disease progression curve)

Broad sense heritability ranged from 0.44 for tuber width to 0.92 for tuber flesh oxidation intensity (Table 7). Heritability estimates were high (> 60) for all the traits studied except for tuber width and plant vigour which recorded moderate values of 0.44 and 0.59, respectively. Genetic advance as a percentage of the mean (GAM) was low (< 10%) for tuber width and yam mosaic virus disease severity scores and moderate (10–20%) for plant vigour, tuber dry matter content and tuber length. Tuber flesh oxidation, fresh tuber yield, tuber weight per plant, average tuber weight and number of tubers per plant recorded high GAM estimates (> 20%).

### Genetic correlation among traits

Fresh tuber yield (t ha^−1^) exhibited a positive significant (*p* < 0.01) genetic correlation with average tuber weight, tuber weight per plant, tuber length and tuber width, while the same correlation was positive but not significant (*p* < 0.05) with number of tubers per plant (Table 8). The relationship between plant vigour and tuber weight per plant, fresh tuber yield, tuber length and tuber width were positive and significant (*p* < 0.01). The correlations between yam mosaic virus disease severity score and tuber weight per plant, fresh tuber yield, tuber length, tuber width, average tuber weight and plant vigour were negative and significant (*p* < 0.01).

**Table 8:** Estimates of genetic correlation coefficients of different traits of 81 white Guinea yam genotypes evaluated for two seasons (2022, 2023) in Nyankpala, Ghana

TRAITS	NTPP	TWP	YLD	ATW	TL	TW	DM	YMV
TWP	0.14							
YLD	0.11	0.88**						
ATW	− 0.46 **	0.42**	0.38**					
TL	0.11	0.58**	0.76**	0.12				
TW	− 0.21	0.48**	0.30*	0.35**	0.10			
DM	0.14	− 0.01	− 0.09	0.09	0.04	− 0.17		
YMV	0.12	− 0.66**	− 0.34**	− 0.27*	− 0.34 **	− 0.37**	0.08	
PV	0.01	0.41**	0.51**	0.03	0.31 **	0.41**	0.11	− 0.27*

NTTP: number of tubers per plant; TWP: tuber weight per plant (kg plant^−1^), YLD: fresh tuber yield (t ha^−1^); ATW: average tuber weight (kg tuber^−1^); TL: tuber length (cm), TW: tuber width (cm), DM: tuber dry matter content (%), YMV: Yam mosaic virus disease severity score (area under disease progression curve)

*significant at 5% probability; **significant at 1% probability level

### Selection options and genetic gain

The genetic merits of the breeding lines were assessed using a selection index that concurrently considered the genotypic values of the seven measured traits. Ranking of the breeding lines based on this multi-trait selection index identified four top-performing genotypes (TDr1700004_014, TDr1700004_113, TDr1700001_112, TDr1700002_090) based on a 5% selection intensity (supplementary Table 1). Three out of these four lines identified by the multi-trait index selection as superior were among the top four highest-yielding lines using the single trait selection strategy for fresh tuber yield alone. Fresh tuber yield of these top-performing lines ranged from 21.3 t ha^−1^ for TDr1700002_90 to 33.9 t/ha for TDr1700004_14 while tuber dry matter content varied from 30.1 to 34.5% for TDr1700004_112 and TDr1700004_113, respectively.

**Fig. 2:**
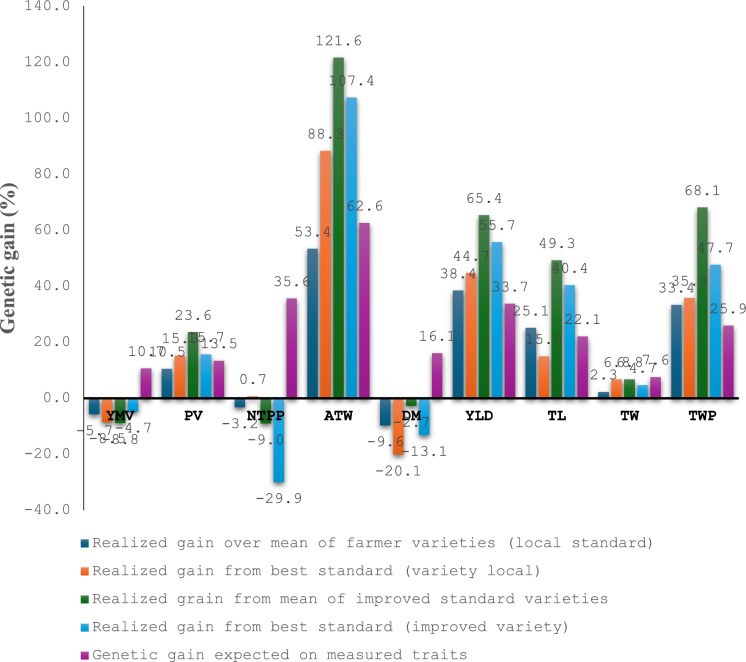
The contrast of expected and realized genetic gains for studied traits with single trait and multiple trait index selection options. YMV: Yam mosaic virus disease severity score (area under disease progression curve), PV: plant vigour, NTTP: number of tubers per plant; ATW: average tuber weight; DM: tuber dry matter content; YLD: fresh tuber yield; TL: tuber length, TW: tuber width, TWP: tuber weight per plant

A comparative analysis of the expected and realized genetic gain for the studied traits was conducted, contrasting multiple traits and single trait selection strategies (Fig. 2). The multiple trait selection index revealed a percentage gain relative to standard local and improved varieties, while the expected selection gain from single trait selection was evaluated as the enhancement in the average value of the selected genotypes over the population mean for each trait. The expected selection gain with single trait selection was low (< 10% of the population mean) for tuber width and moderate (10–20% of the population mean) for yam mosaic virus disease severity, plant vigour, and tuber dry matter content (Fig. 2). In contrast, yield and yield component traits showed high expected genetic gain values (> 20% of the population mean). The multi-trait selection index identified the top 5% performing genotypes, which exhibited positive genetic gain over improved standard and local varieties for plant vigour, tuber length and width, tuber weight per plant, average tuber weight, and fresh tuber yield. Notably, the realized genetic gain was highest for average tuber weight, with a 107.4% and 88.3% increase over the best standard improved variety and local variety, respectively, using the multi-trait selection option. This gain exceeded the expected gain based on single trait selection (62.6%). A similar trend was observed for fresh tuber yield and tuber weight per plant, where the multi-trait selection option yielded higher genetic gain than expected from single trait selection. However, the expected genetic gain with single trait selection was higher than the realized gain for tuber width and tuber length over local varieties.

Contrary to expectations, the multiple trait selection approach yielded a realized genetic gain in an undesirable direction for tuber dry matter content and number of tubers per plant. The mean tuber dry matter content and number of tubers per plant of the top 5% selected individuals were lower than those of the check varieties. A negative realized genetic gain of − 20.1% and − 13.1% was observed for tuber dry matter content relative to the best standard local and best standard improved varieties, respectively. This outcome was unexpected, as the single trait selection strategy had predicted a positive expected gain of 16.1% for tuber dry matter content.

The realized genetic gain for yam mosaic virus disease severity score was negative in comparison with both standard local and improved varieties, indicating that the top 5% identified clones using the multiple trait selection index had lower values for this trait than the checks. It is worth noting that the higher the yam mosaic virus disease severity scores value the more susceptible the genotypes are to yam mosaic virus disease, hence the negative genetic gain for this trait is in the desired direction.

## Discussion

Knowledge of the genetic architecture and genetic gain for key traits are very important for the optimization of breeding programs, assessing progress, and identifying areas for improvement. The significance of the genotype factor of all the traits assessed in this study suggests the presence of ample genetic variability within the population for the traits. The higher average performance of the 71 clones for tuber weight per plant, average tuber weight, tuber length and fresh tuber yield in comparison to the mean of the 10 checks indicates the superiority of the advanced breeding lines for yield and yield components. Contrarily, tuber dry matter content and tuber flesh oxidation of the checks were higher than that of the test lines signifying the superiority of the checks for these traits. The higher performance of the breeding lines could be because of the higher frequency of favourable alleles for these yield and yield component traits emanating from the planned hybridization and selection in the new wave of white Guinea yam genotypes (Darkwa et al. 2020).

The similarity of the TDr and SDr lines for most of the studied traits except tuber length and tuber dry matter content has important implications for yam improvement, in that the TDr lines are full sibs emanating from controlled artificial hand pollination of elite breeding lines while the SDr lines are half-sibs from natural open pollination of local varieties. Male and female yam flowers are often produced on separate plants, necessitating the need to set up different crossing blocks for female plants, with multiple or staggered planting dates to facilitate flowering and hybridization (Mondo et al. 2020). Planned and controlled hybridization in yam is very tedious with a very low percentage of fruit and seed set (Norman et al. 2018; Darkwa et al. 2020; Mondo et al. 2020). Half-sib breeding involving random open pollination among selected parents (unsupervised poly cross) produced a high fruit set and therefore a cost-effective and convenient way to produce many seedlings for selection to complement full-sib breeding (Norman et al. 2018). The present findings of the similarity in performance of the TDr and SDr lines for yam mosaic virus disease resistance and yield and yield contributing traits further affirm the complementarity of controlled hand pollination and natural open pollination to enhance population improvement in yam.

The superiority of the checks and the SDr lines (local varieties and their derivatives) for tuber dry matter content and tuber flesh oxidation in this study cannot be overlooked. The checks and SDr lines had higher mean tuber dry matter content and a lower tendency of tuber flesh oxidative enzymatic browning than the breeding lines. A similar trend was reported by Agre et al. (2023) in their study of popular landraces and elite breeding lines of white Guinea yam in Nigeria, wherein the farmer’s landraces had a higher mean dry matter and low tuber flesh oxidation than the breeding lines. This confirms that the local landraces are a valuable source of alleles for the post-harvest tuber quality traits which are low in the white Guinea yam breeding populations and should therefore be revisited for trait introgression. Corroborating, the present findings, Otoo et al. (2018) also reported a higher tuber yield of advanced breeding lines compared to the local landraces in Ghana.

The superiority of the HIGH lines for plant vigour, yam mosaic virus disease severity, number of tubers per plant, tuber weight per plant, average tuber weight, and tuber length compared to the LOW lines signifies the contribution of these traits to tuber yield and their relevance in identifying high yielding yam genotypes. The results of the genetic correlation among the traits further buttress this point. The genetic correlation between these traits and tuber yield was high and positive, while the negative correlation between the yam mosaic virus disease severity score and all the yield and yield component traits further explains the low yield performance of the LOW lines. The results of the genetic correlation among the traits are similar to the findings of Adewumi et al. (2023) who reported a positive association between tuber yield and plant vigour and a negative correlation between yam mosaic virus disease severity and plant vigour. High yielding white Guinea yam clones can therefore be identified quickly at the vegetative stage based on the plant vigour to facilitate crossing decisions.

Genotypic variance estimates were generally higher than the corresponding environmental variance for the traits studied, signifying the preponderance of the genetic factor in the trait expressions. The genetic factors’ influence in modulating the traits was particularly high for the number of tubers per plant, average tuber weight, tuber flesh oxidation and fresh tuber yield, as shown by the high genotypic coefficient variation values. These traits are therefore under high genetic influence and could be improved by simple phenotypic selection. Traits exhibiting low genotypic coefficient of variation (GCV), such as yam mosaic virus disease severity, tuber width, and tuber dry matter content, demonstrate reduced genetic control. The low GCV values for these traits indicate a limited genetic basis for variation, resulting in restricted phenotypic expression. Consequently, this is reflected in the minimal differences observed in tuber width and tuber dry matter content between the HIGH and LOW lines, as well as the similarities in tuber width and yam mosaic virus disease severity among the TDr and SDr lines, and yam mosaic virus disease severity and tuber width among the lines and checks. This suggests that environmental factors and epigenetic influences may play a more significant role in shaping the expression of these traits, rather than genetic variation. These findings align with previous research by Adewumi et al. (2023) and Olatunji et al. (2024) who reported similarly high broad-sense heritability estimates for yield and yield component traits in white Guinea yam. Traits exhibiting high broad-sense heritability estimates are characterized by a larger proportion of total genetic variance attributed to both additive and non-additive effects, including dominance and epistasis. Broad sense heritability is particularly relevant in clonal crops, such as yam, where vegetative propagation enables the capture and transmission of all genetic effects such as additive, dominance, and epistasis from one generation to the next (Norman et al. 2021). This contrasts with non-clonally propagated crops, where only additive effects are fully transmitted. The high heritability estimates and correspondingly high genetic advance as a percentage of mean observed for number of tubers per plant, average tuber weight, tuber length, tuber flesh oxidation and fresh tuber yield suggest the predominance of additive gene action in modulating these traits and therefore, amendable to fast phenotypic selection for improvement. Tuber dry matter content and yam mosaic virus disease severity score with high broad sense heritability and corresponding moderate and low genetic advance, respectively, may not respond quickly to phenotypic selection for improvement.

The high genetic gain observed for the yield and yield components traits (fresh tuber yield, average tuber weight, tuber weight per plant, number of tubers per plant and tuber length) in this study suggests a higher focus of the breeding program on tuber yield enhancement (Darkwa et al. 2020). The low expected genetic gain for traits such as tuber width and tuber dry matter content, along with the moderate improvement in yam mosaic virus disease severity and plant vigour, aligns with their moderate overall heritability and low estimates of genetic advance. This indicates that these traits are influenced by a more intricate inheritance pattern, making it difficult to achieve significant progress through basic phenotypic selection methods. In agreement with the present findings, Norman et al. (2021) reported higher non-additive genetic variance components for tuber dry matter content and yam mosaic virus disease resistance and corresponding negligible additive genetic variance for these traits compared with fresh tuber yield in white Guinea yam. This suggests the importance of dominance and epistasis in controlling the inheritance of tuber dry matter content and yam mosaic virus disease resistance. Since only the additive genetic effects are transmitted to the progenies through sexual reproduction, Asfaw et al. (2021) suggested that improving the base population by crossing individuals with high additive genetic values would enhance genetic gain for these traits in the breeding population.

The high realized genetic gain obtained for the best 5% lines based on the multi-trait selection index for the yield and yield component traits over both standard local and improved varieties confirms the superiority of the advanced breeding lines. The higher realized genetic gain from the multi-trait index selection strategy observed for most of the traits compared with the single trait selection strategy buttresses the usefulness of index selection in identifying superior yam clones, as also reported by Asfaw et al. (2021); Norman et al. (2021; 2022); Adeyinka et al. (2023) and Ouattara et al. (2024), who also identified superior yam genotypes for economically important traits by applying multiple trait selection criteria.

Despite the importance of dry matter content to the textural characteristics of food products derived from yam and used by consumers to evaluate the acceptability of the product (Otegbayo et al. 2011), tuber dry matter content was reduced with the multi-trait index selection of the best 5% progenies while small progress was attained following the single trait selection strategy. The high genetic gain for tuber yield and yield components and the very low gain for tuber dry matter content observed in this study are in tandem with the findings of Norman et al. (2022), who predicted genetic gain for tuber yield and dry matter content in white Guinea yam using the factor analysis and ideotype-design (FAI-BLUP) index. The current observations further align with Asfaw et al. (2024), wherein historical data was used to assess the trends in genetic gain in the oldest white Guinea yam breeding program. This has significant implications for the yam breeding programs in the region in that a trade-off must be made to ensure a balance between the various economically important traits such that post-harvest food quality is not compromised in improving yield. Implementation of heterosis exploiting breeding schemes or population hybrid breeding could enhance these complex traits in clonal crops (Grüneberg et al. 2009; 2015). Following this approach, Grüneberg et al. (2015) reported significant genetic gains for dry root yield, resistance to sweet potato virus disease and sugar content in sweet potato.

## Conclusion

This study highlighted the genetic control of key economic traits in white Guinea yam wherein both additive and non-additive genetic mechanisms modulated tuber yield traits and dry matter content, respectively. The local landraces or farmer varieties are a valuable source of alleles for the post-harvest tuber quality traits including dry matter content that are lacking in the breeding program hence the need for introgression. The breeding scheme currently being implemented has made giant strides in improving tuber yield but at the expense of dry matter content, necessitating modifications in parental clone selection and crossing plans to improve post-harvest tuber quality traits. The implementation of half-sib breeding resulted in comparable outcomes with the full-sib approach and could therefore complement breeding efforts in white Guinea yam programs.

## Supplementary Information

Below is the link to the electronic supplementary material.

Supplementary file1 (XLSX 24 KB)

## Data Availability

Data available upon request to the corresponding author.
